# Expert Consensus on The Management of Dermatophytosis in India (ECTODERM India)

**DOI:** 10.1186/s12895-018-0073-1

**Published:** 2018-07-24

**Authors:** Murlidhar Rajagopalan, Arun Inamadar, Asit Mittal, Autar K. Miskeen, C. R. Srinivas, Kabir Sardana, Kiran Godse, Krina Patel, Madhu Rengasamy, Shivaprakash Rudramurthy, Sunil Dogra

**Affiliations:** 1 Department of Dermatology, Apollo Hospital, Chennai, India; 2 Department of Dermatology, SBMP Medical College, BLDE Deemed University, Bijapur, India; 3 Department of Dermatology, R.N.T. Medical College and Hospital, Udaipur, India; 4 Dr Miskeen’s Central Clinical Microbiology Lab, Thane, India; 5 Department of Dermatology, PSG Hospitals, Peelamedu, Coimbatore India; 6 Department of Dermatology, Venereology and Leprosy Dr. Ram Manohar Lohia Hospital and Post Graduate Institute of Medical Education and Research, New Delhi, India; 7 Department of Dermatology, Padmashree Dr D Y Patil University, Navi Mumbai, India; 8 Department Of Dermatology, GMERS Medical College & Hospital, Sola, Ahmedabad, India; 9 Department of Dermatology (Mycology), Madras Medical College, Chennai, India; 10 Mycology Division, Department of Medical Microbiology, Postgraduate Institute of Medical Education & Research (PGIMER), Chandigarh, India; 11 Department of Dermatology, Postgraduate Institute of Medical Education & Research (PGIMER), Chandigarh, India; 12 Department of Dermatology, Apollo Hospital, Greams Road No: 21, Greams Lane, Off Greams Road, Chennai, India

**Keywords:** Dermatophytosis, Consensus, Tinea, Delphi, naïve, Recalcitrant, Combination therapy

## Abstract

**Background:**

Dermatophytosis management has become an important public health issue, with a large void in research in the area of disease pathophysiology and management. Current treatment recommendations appear to lose their relevance in the current clinical scenario. The objective of the current consensus was to provide an experience-driven approach regarding the diagnosis and management of tinea corporis, cruris and pedis.

**Methods:**

Eleven experts in the field of clinical dermatology and mycology participated in the modified Delphi process consisting of two workshops and five rounds of questionnaires, elaborating definitions, diagnosis and management. Panel members were asked to mark “agree” or “disagree” beside each statement, and provide comments. More than 75% of concordance in response was set to reach the consensus.

**Result:**

KOH mount microscopy was recommended as a point of care testing. Fungal culture was recommended in chronic, recurrent, relapse, recalcitrant and multisite tinea cases. Topical monotherapy was recommended for naïve tinea cruris and corporis (localised) cases, while a combination of systemic and topical antifungals was recommended for naïve and recalcitrant tinea pedis, extensive lesions of corporis and recalcitrant cases of cruris and corporis. Because of the anti-inflammatory, antibacterial and broad spectrum activity, topical azoles should be preferred. Terbinafine and itraconazole should be the preferred systemic drugs. Minimum duration of treatment should be 2–4 weeks in naïve cases and > 4 weeks in recalcitrant cases. Topical corticosteroid use in the clinical practice of tinea management was strongly discouraged.

**Conclusion:**

This consensus guideline will help to standardise care, provide guidance on the management, and assist in clinical decision-making for healthcare professionals.

## Background

Superficial fungal infections are caused by dermatophytes, non-dermatophytic moulds and commensal yeasts [1]. Dermatophytes, the most common causative agents, are assuming high significance in developing countries like India [1].These organisms metabolise keratin and cause a range of pathologic clinical presentations, including tinea pedis, tinea corporis, tinea cruris, etc. [2] Although usually painless and superficial, these fungi can behave in an invasive manner, causing deeper and disseminated infection and should not be neglected [3]. The lesions may become widespread and may have significant negative social, psychological, and occupational health effects, and can compromise the quality of life significantly [4].

Currently, dermatologists across India are inundated with cases of dermatophytosis presenting with unusual large lesions, ring within ring lesions, multiple site lesions (tinea cruris et corporis), and corticosteroid modified lesions, making diagnosis a difficult bet [5]. This changed face of dermatophytosis has created a real panic among dermatologists. In addition, chronicity of the disease has plagued the patients unlike any other dermatological condition in the country [5]. The recent prevalence of dermatophytosis in India ranges from 36.6–78.4% [6] (Table 1).

**Table 1:** Epidemiology of dermatophytosis in India

Author (Year)	Area	Sample size	Clinical subtype	Predominant dermatophyte isolate	M:F	Common age group affected
Bhatia et al *(*2014) [55]	North India	202	Tinea corporis (39.1%)	*T.mentagyrophyte* (63.5%) *T. rubrum* (31%)	5.7:1	21–50 years
Kucheria et al (2015) [56]	North India	100	Tinea corporis (31%)	*T. rubrum* (46.4%) T. *mentagyrophyte* (30.35%)	1.3:1	21–30 years
Naglot et al (2015) [6]	North-east India	632	Tinea corporis (34.82%)	*T. rubrum* (50.15%) *T. mentagyrophyte* (30.35%)	4.4:1	21–40 years
Putta et al (2016) [57]	West India	80	Tinea corporis (41.25%)	*T.mentagyrophyte* (37.74%) *T. tonsurans* (28.3%)	1.5:1	21–40 years
Ramaraj et al (2016) [58]	South India	210	Tinea corporis (63.27%)	*T. rubrum* (48.95%) *T.mentagyrophyte* (44.75%)	4:3	21–40 years
Gupta et al (2014) [1]	Central India	100	Tinea unguium (52.0%)	*T. rubrum* (41%)	3.7:1	> 60 years

The isolation of the dermatophyte species shows minor geographic variations, as evident in studies conducted in different parts of India (Table 1).

Despite the increasing prevalence of cutaneous dermatophytosis across the world, and especially in the tropics, research in this area has often been neglected; hence it continues to be prevalent worldwide, and poses a therapeutic challenge to practitioners [2]. The American Academy of Dermatology guidelines on the management of tinea cruris and corporis were published two decades ago, while the recent guidelines by the British Association of Dermatology focused only on tinea capitis and onychomycosis [7–9]. Also, the treatment recommendations in the standard textbooks of dermatology appear to have lost their relevance in the current clinical scenario [10]. Thus, the management of dermatophytosis in India is in need of an evidence-based, experience-driven, practical approach from the experts in the field [10, 11]. It was therefore decided to set up an Indian Expert Forum Consensus Group with the objective of laying down recommendations for the diagnosis and management of dermatophytosis in India.

### Issues

The current face of dermatophytosis in India has possibly been an outcome of a complex and intrigued interplay between host, fungus, drug and environment, contributed by multiple factors, including more humid and warmer climate, the absurd use of topical corticosteroid-based combinations, the increased use of broad spectrum antibiotics, the increasing burden of immune-compromised population, the widespread use of antifungals in the agricultural industry, and the questionable role of antifungal drug resistance [10–12].

It is important to recognise that, in India, registries of all diseases, including fungal diseases are not maintained. It is difficult to predict the climatic, geographical or therapeutic changes in the incidence and prevalence of the fungal infection. Much of what is discussed is assumption, which is why creating a consensus is difficult. The theoretical aspects of pharmacokinetics need not match the clinical response to the drug in different individuals. This factor can be decided only with a good registry. These alarming aspects regarding dermatophytosis and their impact on the quality of life, warrant timely address.

### Scope and objectives

Dermatophytosis management has become an important public health issue with a large void in research in the area of disease pathophysiology and management [2]. The existing evidence is primarily based on observational cohort studies rather than randomised controlled trials (RCT). Properly designed RCTs will be required to address these need gaps [10, 11]. There are published guidelines on tinea capitis and unguium [8, 9]. However, these are not applicable for the treatment of other dermatophytosis, like tinea corporis, cruris and pedis, in the current scenario in India.

The scope of this consensus is to bridge this gap and provide an experience-driven approach regarding the diagnosis and treatment for dermatophytosis, including tinea corporis, tinea cruris and tinea pedis.

The consensus was planned around three clinical domains: definitions, laboratory diagnosis and treatment. To our knowledge, this is the first expert consensus developed by the Delphi method for the diagnosis and management of dermatophytosis in India.

This consensus statement was developed using a modified Delphi method - a rigorous process that minimises bias and facilitates a consensual position [13].

## Methods

An invitation to participate in the survey was sent by mail in April 2017, to 14 experts working in the field of clinical dermatology and mycology, selected by lead expert Dr. Murlidhar Rajagopalan, according to their clinical experience, their interest in the field as reflected by their international publications, and further, on their experience in generating guidelines.

**Fig. 1:**
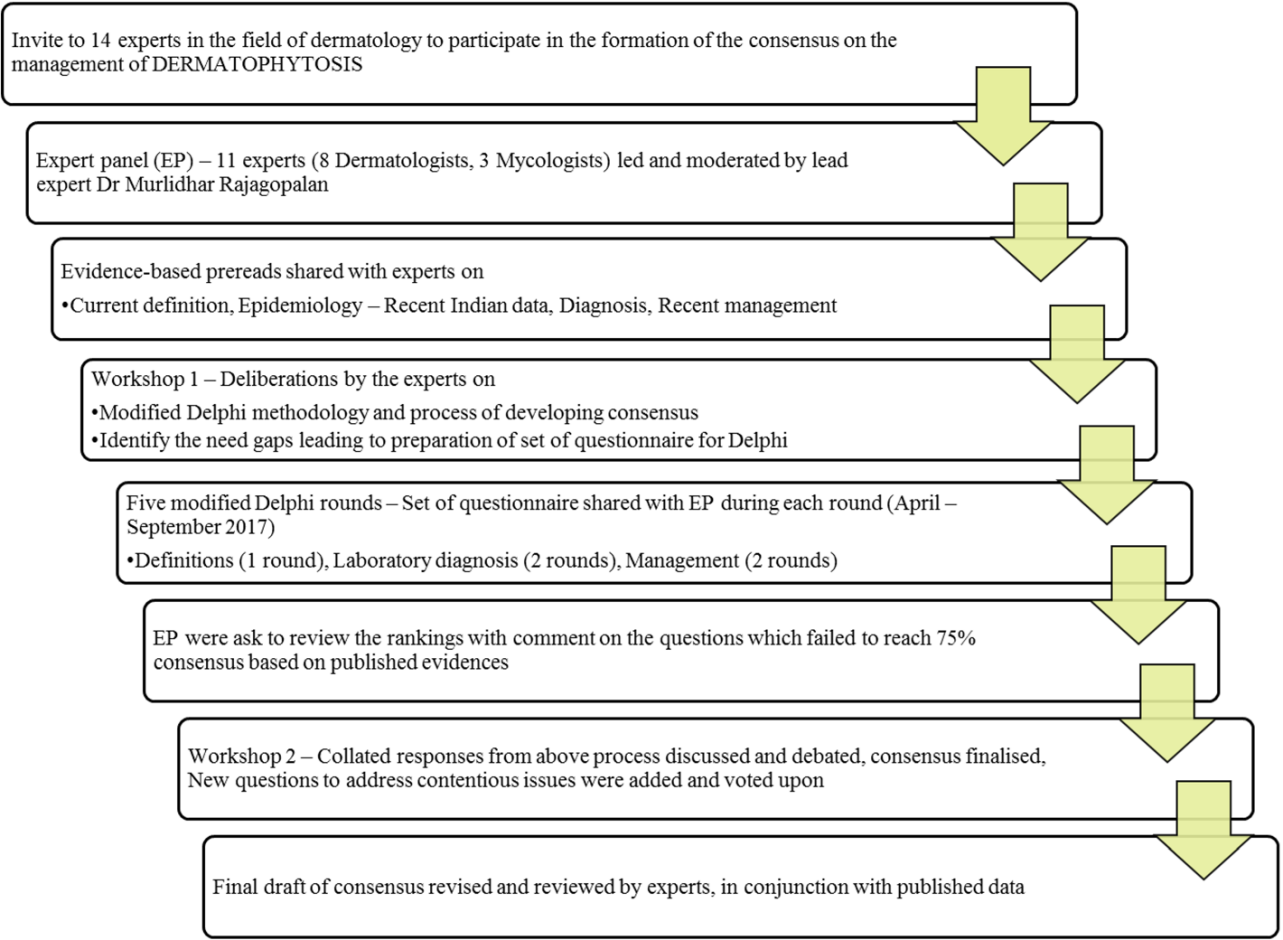
Consensus Workflow

Eleven experts (listed in the appendix) including eight dermatologists, and three mycologists finally participated in five rounds of a web-based modified Delphi Method from April to September 2017, to develop both a consensual statement on the management of dermatophytosis in the current alarming situation of increased incidence, as well as the prevalence of dermatophytosis in India (Fig. 1).

The literature on dermatophytosis was first reviewed using key-words like “tinea infection”, “superficial fungal infections” etc. to retrieve relevant articles on epidemiology, pathophysiology, and management for exploration in the modified Delphi. All experts answered each round (five rounds, 10 questions each) by e-mail. Questions for the rounds were first tested for feasibility and clarity by four non-participants, prior to diffusion to the expert panel (EP). Each Delphi round was delivered by e-mail. E-mail reminders were sent until all members of the EP answered each round of questions. The process was supervised by a lead expert.

The results were analysed after each round, and summary reports describing aggregated group responses were sent to participants in order to allow them to review their answers to the next-round questionnaire. Questions exhibiting a low rate of similar response, after two rounds, were removed to address another field of interest.

The first set of questionnaire was designed to reach a consensus on the definitions for the terminologies including dermatophytosis, chronic dermatophytosis, recurrent dermatophytosis, relapse, trichophytonrubrum syndrome, recalcitrant, and body surface area (BSA). The next two sets of questionnaires were based on a laboratory diagnosis exploring the potential role of KOH mount, fungal culture, dermoscopy and molecular techniques to know their implication in the disease management. The fourth and fifth set of questionnaires were based on understanding the current practice in the management of varied tinea presentations. The participants were also asked to justify their choices.

More than 75% of concordance in response was necessary to reach consensus. Experts arrived at this relatively low figure for consensus based on Delphi after testing the initial questionnaires with very high variability in response. This required restructuring of the questions and redefining what is concordance for the purpose of this survey. However, in the final round of voting when the entire process was reviewed and votometers were used to record opinions on well-defined problems, a concordance of more than 85% was reached in 90% of the responses. Finally, the experts were asked to revise the ranking with written comments on the questions which did not reach the 75% consensus.

## Results

The expert panel first achieved consensus on the definitions for the terminologies, as listed in Table 2, during the first Delphi round.

**Table 2:** Definitions

Term	Definition
Dermatophytosis	Dermatophytosis (ringworm or tinea) is an infection of the skin or skin derivatives, caused by fungi known as dermatophytes leading to erythema, small papules, plaques, vesicles, fissures, and scaling having ring-like morphology. Dermatophytes are filamentous fungi prone to invade and multiply in keratinised tissue, i.e. skin, hair and nails**.**
Naïve infection	A given subject is not previously exposed to a particular infection of a given disease or treatment for that disease.
Chronic Dermatophytosis	Dermatophytosis is considered to be chronic when the patients who have suffered from the disease for more than 6 months to 1 year, with or without recurrence, in spite of being adequately treated.
Recurrent Dermatophytosis	Dermatophytosis is considered to be recurrent when there is re-occurrence of the disease (lesions) within few weeks (< 6 weeks) after completion of the treatment.
Relapse	Relapse denotes the occurrence of dermatophytosis (lesions), after a longer period of infection-free interval (6–8 weeks) in a patient who has been cured clinically.
Trichophyton Rubrum Syndrome	Trichophyton Rubrum Syndrome is defined as, (A) Skin lesions at the following four sites: (1) Feet, often involving soles; (2) Hands, often involving palms; (3) Nails; and (4) At least one lesion in another location other than (1) (2) or (3), except for the groin. (B) Positive microscopic analyses of potassium hydroxide preparations of skin scrapings, in all four locations. (C) Identification of *Trichophyton rubrum* by cell culture at three of the four locations at least. For diagnosis of TRS, the criteria (A) and (B) and (C) have to be fulfilled.
BSA	The area of outstretched palm from the wrist to the tip of the fingers can be considered roughly 1% of the body surface area. Less than 3% can be counted mild, 3–10% as moderate, and more than 10% as severe, in terms of the extent of involvement.

For practical purposes, experts suggested the use of the following terminologies:

**Recalcitrant tinea infection:** This is a generic term that may refer to relapse, recurrence, re-infection, persistence of infection, and chronic infection.

BSA as a clinical assessment tool for dermatophytosis can be a novel concept in defining the severity of the lesions, as shared by all EP members. The application of BSA as a tool in clinical practice will require a further backup through well-designed RCTs.

Questionnaires for Delphi rounds 2 and 3 were based on laboratory diagnoses, which were shared through email to all EP members, post the response to Round 1.

For optimising the laboratory results, the quantity and quality of the material for examination are critical, as agreed by all EP members. Eighty percent of the members agreed for the collection of specimen from the edge of the lesion, as viable hyphae are seen more near the edge of lesion. Scalpel blades and blunt dermal curretes should be used to collect the sample, and it should be transported in dry black strong paper, to avoid the bacterial contamination.

The point of care test recommended by the panel for confirming the diagnosis of dermatophytosis was 10% KOH mount of skin scraping. Further, the EP panel commented on observing the KOH mount, 15–30 min after preparation, to improvise the sensitivity. The adequacy of the sample, and the appropriateness of the collecting tool and expertise will decide the sensitivity and specificity of the diagnosis.

Even though the fungal culture is gold standard in the diagnosis of dermatophytosis, experts were against its routine use in clinical practice to confirm the diagnosis. But fungal culture should be considered in recalcitrant and multisite tinea (tinea cruris et corporis) cases.

EP members identified dermoscopy as an adjunctive tool for the management of dermatophytosis, highlighting the involvement of vellus hair on dermoscopy examination, as an indicator for systemic therapy.

MALDI-TOF (matrix-assisted laser desorption ionisation-time of flight) mass spectrometry (MS was perceived as a promising experimental technique, and not as a practical tool in the real world by all the experts. A prerequisite of culture is mandatory while considering MALDI-TOF, as it cannot be performed on direct clinical samples. Diagnostic tools for tinea unguium and tinea capitis were out of the scope of this discussion.

Questionnaires for Delphi rounds 4 and 5 were based on the management of dermatophytosis, which were shared through email to all EP members, post the response to Round 3.

Experts highlighted the importance of factors, including the site of the infection, the skin area involved (dry/sebum rich), previous antifungal exposure, and the age of the patients while choosing antifungal therapy.

Interdigital is the most common presentation of tinea pedis. The reservoir effect of tinea pedis and its role in the infections of other anatomical sites were emphasised by the members. Bacterial coinfection is commonly associated with tinea pedis, and occasionally found in cases of tinea cruris and corporis. A majority of the experts recommended the use of combination (topical and systemic) antifungal therapy, as empiric treatment in the management of naïve and recalcitrant cases of tinea pedis. Experts favoured the use of topical azoles in tinea pedis management as many non-dermatophyte species cause tinea pedis. However, they believed that the choice of the topical antifungal is also influenced by the clinical subtype of the disease, e.g. ciclopiroxolamine in the management of recalcitrant tinea pedis. In case of systemic antifungal agents, experts favoured terbinafine (250 mg once daily) in naïve cases of tinea pedis whereas itraconazole (200 mg - 400 mg/day, in divided dose) was preferred in recalcitrant and severe cases. The minimum duration of the treatment should be 2–4 weeks in naïve tinea pedis and more than 4 weeks in recalcitrant cases.

The majority of the experts recommended the use of topical therapy in the management of naïve cases of tinea cruris and corporis (localised lesion) while combination therapy is recommended in recalcitrant tinea cruris. However, the choice of topical antifungal agents varied according to the region and personal experience of the individuals. Experts also commented on the fact that in case of naïve tinea corporis with extensive skin involvement or lesions with papules, combination therapy should be favoured. Experts recommended that topical azoles should be the empiric agent of choice in the management of naïve and recalcitrant cases, while no consensus was formed for systemic antifungal agent of choice. In case of systemic antifungal agents, experts preferred either terbinafine (250 mg once daily) or itraconazole (100 mg – 200 mg/day) in naïve cases whereas itraconazole (200 mg - 400 mg/day) was preferred in recalcitrant cases. The minimum duration of the treatment should be 2–4 weeks in naïve tinea cruris and more than 4 weeks in recalcitrant cases.

In case of tinea incognito, where corticosteroids had been used, experts recommended abrupt stoppage of corticosteroids except in settings of steroids induced rosacea, where it is withdrawn in few days. The panel recommended Itraconazole 100 mg–200 mg, twice daily, for the treatment of tinea incognito. The duration of the therapy should be 4–6 weeks or more, in tinea incognito.

Experts recommended that the treatment should be continued for 2 weeks, post clinical cure for topical agents, whereas systemic therapy should be continued in recalcitrant cases only.

Looking at the current explosion of dermatophytosis in India, experts unanimously rejected the role of topical corticosteroid in the management of dermatophytosis.

Doubling of the dose in case of systemic antifungal agents is not required in case of naïve tinea cases, while in the case of recalcitrant tinea infections, doubling the dose is strongly favoured for terbinafine (500 mg/day), while a consensus could not be reached for doubling the dose of itraconazole.

Though there are multiple agents used as supplemental treatment for tinea infections, the role of 6% salicylic acid, antihistamines and moisturisers was agreed upon by the experts. However, these agents are not recommended in all cases. Bacterial super infections need to be treated with appropriate antibacterial agents.

Baseline liver function tests (LFTs) and periodic monitoring are required before starting the systemic antifungal therapy in recalcitrant cases, and in the elderly, especially with prolonged use of itraconazole, while it is not mandatory in naïve cases.

In the paediatric age group, there was no specific recommendation for topical antifungal agents, but fluconazole and terbinafine were preferred as the systemic choice of agents. In the case of pregnant females, topical agents should be preferred, while systemic therapy should be avoided, as far as possible.

## Discussion

As discussed earlier, the standard recommendations from current guidelines are no longer relevant in the current Indian context [10]. Hence it was agreed mutually between the experts of clinical dermatology and mycology to develop the experience-based consensus statement.

There are no standard definitions for the various terminologies like relapse, recurrence, persistence and chronic infections, which add to the confusion in the management of dermatophytosis in real world settings. Through Delphi process, experts could arrive at workable explanations of various terminologies for better understanding the clinical profile of dermatophytosis (Table 2).

### Laboratory diagnosis

The evolving clinical presentation poses difficulty of clinical differentiation of dermatophytosis from other non-mycotic dermatitis. This often necessitates a laboratory diagnosis to initiate appropriate treatment [14, 15]. As shown in the results, the quality and quantity of the clinical sample are imperative for isolation of dermatophytes as reappraised by Pihet et al. in a recent review [15]. For better yield of results, the edge of the lesion is the most prolific site for skin scrapings [15, 16]. This is in accordance with the current Delphi results. Various instruments were suggested in literature for collecting skin scrapings like scalpel blades, dermal blunt curretes or edge of slide [15–17]. However, based on the Delphi results, experts did not favour any specific instrument for sample collection.

The point of care test for the rapid detection of dermatophytosis is microscopic examination of 10% KOH mount of skin scrapings, as agreed upon by the experts. The importance of KOH as a simple, rapid, inexpensive and efficient screening technique was highlighted previously by Kurade et al., Pihet et al., McKay et al. [15–17] Hence it is advisable to perform a microscopic examination of 10% KOH mount of skin scrapings in every case, for a better treatment outcome.

Fungal culture provides the definitive identification of fungal species, but its routine application is deferred as it often lacks the sensitivity, prolonged turnaround time (TAT) and paucity of availability [15, 18]. The experts were of the same opinion. However, they recommended the use of culture in special situations, including recalcitrant and multisite tinea (tinea cruris et corporis) cases.

Therapeutic implication of the involvement of vellus hair was recognised by Gomez – Moyano et al. in their large series on tinea of vellus hair [19]. The expert panel also recognised the importance of the identification of vellus hair involvement by dermoscopy and the role in management, as reflected in the consensus shown in the results.

L’Ollivier C et al. recently highlighted the role of the MALDI-TOF MS procedure as a first-line, accurate, economical and faster identification technique for clinical dermatophyte species in routine laboratory [20]. The expert mycologist felt that the routine use of MALDI-TOF MS may not always help in the management as culture is a prerequisite and is available only at a few tertiary level centres across India.

The therapeutic implication of knowing dermatophyte antifungal sensitivity was well recognised by the experts. The current recommendations by “Clinical & Laboratory Standards Institute (CLSI)” lack the consistent correlation between in vitro antifungal sensitivity data and clinical outcome, with lack of MIC breakpoints to categorise the isolate as susceptible, intermediate, or resistant to a particular antifungal agent [21]. Hence, the routine use of antifungal sensitivity is not feasible in real world settings [21].

The clinical appearance of lesions with a history of prior treatment, along with the knowledge of pharmacological properties of antifungal agents will help guide the choice of therapy [22]. Further to this, experts have identified the skin area involved (dry/sebum-rich) and the age of the patients, as additional factors influencing the choice of treatment. An ideal topical treatment should have a high cure rate, low relapse rate, and short duration of action, and should cause minimal adverse effects. In addition, it is important to find a treatment regimen that is satisfactory to the person with the condition, to ensure compliance.

### Tinea pedis

Tinea pedis usually begins from interdigital spaces with patterns like hyperkeratotic dry, scaly, macerated, oozing and erosive lesions [23]. Clinical pattern of tinea pedis is not pathogen specific since many non-dermatophyte species are recognised as etiological agents of tinea pedis [23]. Topical therapy is the mainstay treatment option in patients with tinea pedis [2, 24–26]. The necessity to treat tinea pedis topically arises from the fact that interdigital maceration, fissures and desquamation of the stratum corneum may serve as portals of entry for secondary bacterial infections and also as a reservoir for dermatophytosis of other sites as agreed upon by the experts [27, 28]. In macerated, erosive interdigital tinea pedis, often complicated by secondary bacterial infection, antimycotic solutions, gels, or sprays are preferable. By contrast, a cream or ointment is preferable for the treatment of dry and scaly hyperkeratotic tinea pedis [29]. Agents with broad-spectrum antimycotic activity covering dermatophytes, yeasts and molds need to be used in tinea pedis as suggested by the experts [30]. In addition to their antimycotic effects, imidazole also exhibits good antimicrobial effects against Gram-positive bacteria and favoured as the choice of agent by the experts [29, 30]. Other topical agents which are useful in tinea pedis are allylamines, ciclopiroxolamine, amorolfine, etc.

As per Cochrane review systemic therapy is usually used for chronic or failure of topical therapy in tinea pedis [31]. Systemic therapy is also preferable in severe disease forms, such as moccasin and hyperkeratotic tinea pedis [29]. The current therapeutic regimen includes terbinafine 250 mg daily for 2 weeks, or itraconazole 200 mg daily, for 4 weeks [2, 29, 30]. Looking at the current scenario, experts favoured the role of combination therapy in all patients with tinea pedis. However, there are no comparative studies available on the combination of systemic and topical therapy versus monotherapy [2]. Experts commented that while using combination therapy, drugs from different classes should be preferred for wider coverage and to prevent emergence of resistance. In naïve cases, terbinafine 250 mg/day should be preferred while in recalcitrant cases or severe disease forms, itraconazole 200 mg – 400 mg/day in divided dose is the drug of choice. According to experts, the minimum duration of therapy in naïve tinea pedis should be 2–4 weeks, while in case of recalcitrant cases it should be 4 weeks.

### Tinea cruris and corporis

As the dermatophytes causing tinea cruris and corporis infection are limited to the superficial keratinised tissue, topical treatments are the most appropriate to use in patients with naïve tinea cruris and corporis, provided the infection is not widespread [32, 33]. Experts were of the same opinion, and recommended the use of topical antifungal agents in naïve cases. The superiority of one class of topical antifungal over another has not been well established in clinical trials [34]. However, looking at the current scenario, experts favoured the use of topical azoles over allylamines in virtue of antibacterial, anti-inflammatory and broad spectrum antimycotic properties [33]. Topical antifungal treatments are normally well-tolerated and tend not to cause adverse effects.

Extensive superficial lesions or lesions with papules and pustules require oral therapy [2, 35–37]. As discussed earlier, experts favoured the use of combination therapy in such patients and patients with recalcitrant infection. According to experts, terbinafine (250 mg once daily) and itraconazole (100 mg – 200 mg/day) are equally effective in treating naïve cases. In case of recalcitrant cases, experts recommended the use of itraconazole (200 mg – 400 mg/day, in divided dose) along with appropriate topical therapy. According to experts, the minimum duration of therapy in naïve cases should be 2–4 weeks, while in recalcitrant cases, it should be 4 weeks.

Tinea cruris, in most cases, results from autoinoculation in patients with pre-existing tinea pedis [23]. Concomitant tinea pedis, if present, should be treated to reduce risk for recurrence [38]. Other interventions that may be helpful include daily use of desiccant powders in the inguinal area and avoidance of tight-fitting clothing and non-cotton underwear [10, 39]. The role of examining and treating close contacts, and avoidance of body contact sports, were also emphasised as an important input to be counselled when treating a patient with tinea infections.

### Tinea incognito

Tinea incognito is a mycotic infection of the skin that has been modified by improper use of steroids and topical immunomodulators such as calcineurin inhibitors in a way that renders it no longer diagnostic [40, 41]. As some high-potency topical steroids are easily accessible as over-the-counter (OTC) products and non-dermatologists can also prescribe topical steroids freely without any fungal examination, the incidence of this form of tinea seems to be gradually increasing [41]. A classic feature is that the inflammatory lesion and the formation of scales may be suppressed, but symptoms relapse when application of the steroid creams is stopped. Alternatively, the lesions may present as marked purulent folliculitis and a diffuse inflammatory response [40–42]. Experts believe that in tinea incognito, oral antifungal therapy is essential and topical corticosteroids should be stopped abruptly. Itraconazole 200 mg – 400 mg daily, for 4–6 weeks or longer, should be the drug of choice as per experts.

### Role of topical corticosteroids

A global expert panel meeting on the topical treatment of superficial dermatophytoses, by reviewing numerous meta-analyses, arrived at the conclusion that corticosteroid-based combination therapy has an important role in inflammatory dermatophytosis [43]. Though the experts recognised that corticosteroids may have some role in inflammatory dermatophytosis, looking at the current scenario they vetoed the use of topical corticosteroids in any type of dermatophytosis in India. The experts felt that this would give impetus to prescription of steroids in infective dermatoses, a problem which is already plaguing the country.

### Practice recommendations

As per the recommendation in the American Academy of family physicians (AAFP), topical antifungals should be continued for at least 1 week post clinical resolution [22]. However, experts recommended that topical antifungal agents should be continued for 2 weeks post clinical cure, which is in accordance with the recent review on the current scenario of dermatophytosis in India [44]. The continuation of systemic therapy for 2 weeks after clinical resolution in recalcitrant cases, is also recognised by the experts. Since some systemic antifungal drugs can cause hepatotoxicity, it is advisable to do baseline LFTs to rule out impaired liver function and periodic follow-up, if the treatment duration exceeds 4 weeks.

### Systemic antifungals in current context

With the current situation of dermatophytosis in India, a radical change in prescription practices has been observed. A majority of dermatologists in India are using a combination of oral antifungals, higher doses of antifungals [30, 45], a longer duration of treatment, and other therapies not even approved for dermatophytosis, for the management of recalcitrant cases, and these tend to benefit the individual patients more [10, 11, 30]. Experts are of same opinion and further recognised the role of a higher dose of terbinafine, however deserted the use of high dose itraconazole due to its non-linear pharmacokinetics.

Experts felt the need to use other systemic antifungals like Griseofulvin (250 mg – 500 mg twice daily) and fluconazole (150 mg–300 mg/week) in patients with whom treatment with terbinafine or itraconazole had failed. However, the delayed clinical response time, a requiring longer duration of the therapy, should be considered before starting the therapy [46].

### Adjuvant therapy

Dermatophytoses are usually associated with several-fold increase in epidermal cell proliferation, leading to epidermal thickening with hyperkeratosis and scaling of the skin [47]. As scales impede the absorption of topical antifungal drugs, the sole use of topical antifungal agents may be ineffective, especially in recalcitrant cases [48]. Keratolytics, by their dual effect, can help in increasing levels of topical antifungals, and removing the stratum corneum where fungi lie [30, 38]. Experts recognised these pathological features and recommended the use of topical salicylic acid 3–6%, as it causes softening of the horny layer and the shedding of scales, but it is not to be used in intertriginous areas or the face.

In dermatophytosis, there is significant increase in transepidermal water loss and specific ultrastructural changes, such as disturbed formation of extracellular lipid bilayers leading to disturbed skin barrier function [47]. This may lead to chronicity of the disease. Considering these facts, experts suggested the use of moisturisers as adjuvant therapy in the management of dermatophytosis. Pruritus being the common symptom of dermatophytosis, experts justified the use of antihistamines, as an adjuvant therapy in acute cases.

### Elderly patients

In elderly patients, the treatment should be individualised. The patient’s need, site and extent of involvement, the presence of comorbidities and the possibility of drug interactions should be considered before starting the treatment [39, 49]. A healthy elderly patient can be treated as per recommendations applied to a young adult. Topical therapy should be favoured in elderly patients; systemic therapy is required only in cases of the failure of topical therapy, extensive lesions and recalcitrant cases. Since systemic triazole drugs (itraconazole, fluconazole) are capable of multiple drug interactions, oral terbinafine should be preferred [39, 49].

### Paediatric age group

Dermatophytosis is relatively less common in the paediatric age group. In one Indian study, only 3.1% prevalence of dermatophytosis has been reported [50]. However, in recent years, an exponential increase in dermatophytosis in the paediatric age group has been noted [39]. Experts favoured the use of topical agents in this age group, owing to rapid turnover of skin, which may contribute to a relatively better clinical response to topical therapy alone. Systemic therapy is advised only in extensive lesions or recalcitrant cases. Experts recommended use of fluconazole and terbinafinein paediatric age group. While fluconazole can be used during infancy, terbinafine is recommended only after 2 years of age.

### Pregnant females

Topical antifungals are minimally or not absorbed systemically, and therefore can be prescribed at any stage of pregnancy [39, 51–53]. Regarding systemic therapy, terbinafine is pregnancy category B, however, data on its use in pregnancy is not present; also, whether terbinafine crosses the placental barrier is unknown [39, 50]. Other systemic antifungals should be avoided during pregnancy. Though clinical studies with itraconazole have not detected any increased risk during pregnancy, considering the risk conveyed by the azole family in humans, the drug should still be avoided during pregnancy [51]. Effective contraception for 2 months, after taking oral itraconazole before conception, is suggested [54]. The experts discussing the consensus agreed with these recommendations without change.

### General measures

Stress on the importance of regularity of medication and adherence to the advice of the physician. Avoid use of tight clothing. Sharing of bed linen, towels and clothes should be avoided. Undergarments, socks, and caps should be regularly washed and dried in the sun and ironed. Patients should be assessed for associated conditions like excessive sweating or obesity which may lead to recurrence. Hence in such patients, frequent change of clothing, use of absorbent powders and deodorants (decrease perspiration), and weight loss should be encouraged.

In case of tinea pedis, medicated powders can be used prophylactically. Use of occlusive footwear and use of slippers in public washrooms should be avoided. Foul smelling and macerated lesions point towards secondary bacterial infection, and should be treated appropriately, using either systemic or topical antibacterial agent.

Entire management pearls are summarised in Table 3.

**Table 3:** Dermatophytosis (Tinea Corporis, Cruris and Pedis) management pearls in Indian settings

Diagnosis	
1. Microscopic examination of 10% KOH mount should be the point of care testing for dermatophytosis. a) Skin scrapings should be collected from the edge of the lesions. b) Transportation should be in dry black strong paper. 2. Sensitivity and specificity of diagnosis depend on a) Adequacy of the sample b) Appropriateness of the sample collection c) Personnel expertise 3. Fungal culture should be reserved in a) Recalcitrant and multisite tinea cases. 4. Dermoscopy examination helps to delineate vellus hair involvement a) Vellus hair involvement requires systemic therapy.	
Management	
1. The choice of the antifungal depends on a) Pharmacological properties b) History of prior exposure to antifungals c) The site and extent of the lesion d) Skin area involved (dry/sebum rich), and the age of patient 2. Naive and recalcitrant tinea pedis cases to be treated empirically with a combination of topical and systemic antifungals. 3. Naïve tinea cruris and corporis (localised lesion) cases to be treated empirically with topical antifungals alone. For extensive lesions and recalcitrant cases, a combination of topical and systemic antifungals should be used. 4. Topical azoles should be the drug of choice, since they exert anti-inflammatory, antibacterial and broad spectrum antimycotic activity. 5. Preferred systemic agents for naïve tinea cases are terbinafine 250 mg daily or itraconazole 100 mg–200 mg daily, and in recalcitrant cases, itraconazole 200 mg–400 mg daily. A higher dose of systemic antifungals can be considered in certain cases including deep inflammatory, multisite lesions, non-responders, T. rubrum syndrome. 6. The minimum duration of treatment should be 2–4 weeks in naïve cases and > 4 weeks in recalcitrant cases. 7. Systemic therapy should be considered in villous hair involvement. 8. Abrupt withdrawal of corticosteroids should be practised in tinea incognito, with Itraconazole, 200 mg – 400 mg daily, for a minimum duration of 4–6 weeks or more. 9. Topical corticosteroid use in clinical practice of tinea management is strongly discouraged. 10. Adjuvant therapies like antihistamines, salicylic acid and moisturisers play important role in the management. 11. Baseline LFTs and periodic monitoring to be considered during systemic therapy and the elderly. 12. Empiric therapy of choice in paediatric age group is topical antifungals alone. Systemic agents like fluconazole and terbinafine to be reserved for extensive lesions and recalcitrant cases. 13. In the elderly, and patients with comorbid conditions, the treatment should be individualised. 14. In pregnancy, topical antifungals are the agents of choice in any trimester.	
Management of Trichophyton Rubrum Syndrome	
1. Identify predisposing host environmental factors 2. Establish the diagnosis: a) Clinically (Involvement of two or more noncontagious sites, hands, feet, nails, absence of deeper lesions) b) Investigation: KOH positivity from all sites, culture positive from at least one site 3. Check for factors such as concomitant HIV infection, use of immunosuppressive etc. Their presence may suggest other diagnosis. 4. Antifungals are to be used for a longer period, and can go up to 3 months. Sometimes They may have to be combined with other antifungals. Some options are: a. Itraconazole 200 mg/ day, for 4–6 weeks. Therapy may be extended till complete clinical resolution. b. Combination of Itraconazole 200 mg/day and Terbinafine 250 mg/day for 4–6 weeks or extended periods. c. Itraconazole 200 mg twice a day × 7 days/month, for 3–5 months, depending on the clinical response. d. Topical Luliconazole/Sertaconazole once/twice a day, for 6 weeks or Topical Terbinafine/Amorolfine, twice daily, for extended periods. 5. Taking care of fomites/household contacts. 6. Fungal Culture and antifungal susceptibility tests, if facility is available. 7. If nails are involved, onychomycosis should be suspected and treated accordingly. 8. Assuring patient compliance for the need of continuous therapy till complete clearance of infection from all sites, use of a topical drug in a proper manner and quantity, etc.	

## Conclusions

Although our work has been an attempt to bridge the gaps between the existing recommendations against the current problem in the field of dermatophytosis, in future, the maintenance of registry, the measurement of herd immunity, measuring skin levels of drug and correlation with blood levels, and response to therapy using different dose schedules with special reference to special situations like recalcitrant, relapse, immunocompromised states or patients with comorbidities will be useful for improving therapeutic outcomes.

Priorities for future research to improve the outcome of dermatophytosis management:

- Improved diagnostic tests, with high accuracy, rapid turnaround time, and prognostic value like BSA that can guide antifungal therapy in real time.
- Direct detection of species causing infection and antifungal resistance from clinical specimen.
- Better risk prediction models, including genetic risk factors to target surveillance and prophylaxis.
- Mechanisms to ensure the attainment of maximal antifungal effect as quickly as possible (e.g. combination therapy, therapeutic drug monitoring).
- Novel immunomodulatory treatments to maximise antifungal effect and minimise immune-mediated damage.
- Collaborative national and international programmes for dermatophytosis management.

## References

[1] Gupta CM, Tripathi K, Tiwari S, Rathore Y, Nema S, Dhanvijay AG (2014). Current trends of Clinico mycological profile of Dermatophytosis in Central India. IOSR-JDMS.

[2] Sahoo AK, Mahajan R (2016). Management of tinea corporis, tinea cruris, and tinea pedis: a comprehensive review. Indian Dermatol Online J.

[3] Bristow IR, Spruce MC (2009). Fungal foot infection, cellulitis and diabetes: a review. Diabet Med.

[4] Jerajani H, Janaki C, Kumar S, Phiske M (2013). Comparative assessment of the efficacy and safety of Sertaconazole (2%) cream versus terbinafine cream (1%) versus Luliconazole (1%) cream in patients with Dermatophytoses: a pilot study. Indian J Dermatol.

[5] Dogra S, Narang T (2017). Emerging atypical and unusual presentations of dermatophytosis in India. Clin Dermatol Rev.

[6] Naglot A, Shrimali DD, Nath BK, Gogoi HK, Veer V (2015). Recent trends of Dermatophytosis in Northeast India (Assam) and interpretation with published studies. Int J CurrMicrobiol App Sci.

[7] Drake LA, Dinehart SM, Farmer ER, Goltz RW, Graham GF, Hardinsky MK (1996). Guidelines of care for superficial mycotic infections of the skin: tinea corporis, tinea cruris, tinea faciei, tinea manuum, and tinea pedis. Guidelines/Outcomes Committee American Academy of Dermatology. J Am Acad Dermatol.

[8] Ameen M, Lear JT, Madan V, MohdMustapa MF, Richardson M (2014). British Association of Dermatologists’ guidelines for the management of onychomycosis 2014. Br J Dermatol.

[9] Fuller LC, Barton RC, MohdMustapa MF, Proudfoot LE, Punjabi SP, Higgins EM (2014). British Association of Dermatologists’ guidelines for the management of tinea capitis 2014. Br J Dermatol.

[10] Verma S, Madhu R (2017). The great Indian epidemic of superficial dermatophytosis: an appraisal. Indian J Dermatol.

[11] Dogra S, Uprety S (2016). The menace of chronic and recurrent dermatophytosis in India: is the problem deeper than we perceive?. Indian Dermatol Online J.

[12] Zhan P, Liu W (2017). The changing face of Dermatophytic infections worldwide. Mycopathologia.

[13] Mitchell RB, Hussey HM, Setzen G, Jacobs IN, Nussenbaum B, Dawson C (2013). Clinical consensus statement: tracheostomy care. Otolaryngol Head Neck Surg.

[14] Hainer BL (2003). Dermatophyte infections. Am Fam Physician.

[15] Pihet M, Le Govic Y (2017). Reappraisal of conventional diagnosis for dermatophytes. Mycopathologia.

[16] Kurade SM, Amladi SA, Miskeen AK (2006). Skin scraping and a potassium hydroxide mount. Indian J Dermatol VenereolLeprol.

[17] McKay M, Walker HK, Hall WD, Hurst JW (1990). Office techniques for dermatologic diagnosis. Clinical methods, the history, physical, and laboratory examinations.

[18] Feuilhade de Chauvin M (2005). New diagnostic techniques. J Eur Acad Dermatol Venereol.

[19] Gómez-Moyano E, Crespo-Erchiga V (2010). Tinea of vellus hair: an indication for systemic antifungal therapy. Br J Dermatol.

[20] L'Ollivier C, Cassagne C, Normand AC, Bouchara JP, Contet-Audonneau N, Hendrickx M (2013). A MALDI-TOF MS procedure for clinical dermatophyte species identification in the routine laboratory. Med Mycol.

[21] Yadav A, Urhekar AD, Mane V, Danu MS, Goel N, Ajit KG (2013). Optimization and isolation of dermatophytes from clinical samples and in vitro antifungal susceptibility testing by disc diffusion method. Res Rev.

[22] Weinstein A, Berman B (2002). Topical treatment of common superficial tinea infections. Am Fam Physician.

[23] Nenoff P, Krüger C, Schaller J, Ginter-Hanselmayer G, Schulte-Beerbühl R, Tietz HJ (2014). Mycology - an update part 2: dermatomycoses: clinical picture and diagnostics. J Dtsch Dermatol Ges.

[24] Crawford F, Hollis S (2007). Topical treatments for fungal infections of the skin and nails of the foot. Cochrane Database Syst Rev.

[25] Durdu M, Ilkit M, Tamadon Y, Tolooe A, Rafati H, Seyedmousavi S (2017). Topical and systemic antifungals in dermatology practice. Expert Rev Clin Pharmacol.

[26] Moriarty B, Hay R, Morris Jones R (2012). The diagnosis and management of tinea. BMJ.

[27] MargaridoLda C (2014). Oral treatments for fungal infections of the skin of the foot. Sao Paulo Med J.

[28] Müller DP, Hoffmann R, Welzel J (2014). Microorganisms of the toe web and their importance for erysipelas of the leg. J Dtsch Dermatol Ges.

[29] Nenoff P, Krüger C, Paasch U, Ginter-Hanselmayer G (2015). Mycology - an update part 3: dermatomycoses: topical and systemic therapy. J Dtsch Dermatol Ges..

[30] Sardana K, Mahajan K, Mrig PA. Fungal Infections: Diagnosis and treatment. First edition, CBS Publishers and Distributors; 2017. p 266–317.

[31] Bell-Syer SE, Khan SM, Torgerson DJ (2012). Oral treatments for fungal infections of the skin of the foot. Cochrane Database Syst Rev.

[32] El-Gohary M, van Zuuren EJ, Fedorowicz Z, Burgess H, Doney L, Stuart B et al. Topical antifungal treatments for tinea cruris and tinea corporis. Cochrane Database Syst Rev 2014;(8):CD009992. http://cochranelibrary-wiley.com/doi/10.1002/14651858.CD009992/full. 10.1002/14651858.CD009992.pub2 PMC11198340 25090020

[33] Van Zuuren EJ, Fedorowicz Z, El-Gohary M (2015). Evidence-based topical treatments for tinea cruris and tinea corporis: a summary of a Cochrane systematic review. Br J Dermatol.

[34] Rotta I, Ziegelmann PK, Otuki MF, Riveros BS, Bernardo NL, Correr CJ (2013). Efficacy of topical antifungals in the treatment of dermatophytosis: a mixed treatment comparison meta-analysis involving 14 treatments. JAMA Dermatol.

[35] Lesher JL (1999). Oral therapy of common superficial fungal infections of the skin. J Am Acad Dermatol.

[36] Bourlond A, Lachapelle JM, Aussems J, Boyden B, Campaert H, Conincx S, Decroix J, Geeraerts C, Ghekiere L, Porters J, Speelman G, Tennstedt D, Kint T, Vandaele R, Haute V, Lint L, Willocx D (1989). Double-blind comparison of Itraconazole with Griseofulvin in the treatment of tinea Corporis and tinea Cruris. Int J Dermatol.

[37] Cole GW, Stricklin G (1989). A comparison of a new oral antifungal, terbinafine, with griseofulvin as therapy for tinea corporis. Arch Dermatol.

[38] Goldstein AO, Goldstein BG. Dermatophyte (Tinea) Infections. Post TW, ed. UpToDate. Waltham: UpToDate Inc. http://www.uptodate.com/contents/dermatophyte-tinea-infections?source=search_result&selectedTitle=1%7E150. Assessed 10 Oct 2017.

[39] Kaul S, Yadav S, Dogra S (2017). Treatment of dermatophytosis in elderly, children, and pregnant women. Indian Dermatol Online J.

[40] Solomon BA, Glass AT, Rabbin PE (1996). Tinea incognito and “over-the-counter” potent topical steroids. Cutis.

[41] Dutta B, Rasul ES, Boro B (2017). Clinico-epidemiological study of tinea incognito with microbiological correlation. Indian J Dermatol Venereol Leprol.

[42] Jacobs JA, Kolbach DN, Vermeulen AH, Smeets MH, Neuman HA. Tinea incognito due to Trichophyton rubrum after local steroid therapy. Clin Infect Dis. 2001;33:142-4. 10.1086/338023 11702294

[43] Schaller M (2015). Dermatomycoses and inflammation: the adaptive balance between growth, damage, and survival. J Mycol Med.

[44] Verma S, Madhu R (2017). The great Indian epidemic of superficial dermatophytosis: an appraisal. Indian J Dermatol.

[45] Babu PR, Pravin AJ, Deshmukh G, Dhoot D, Samant A, Kotak B (2017). Efficacy and safety of terbinafine 500 mg once daily in patients with dermatophytosis. Indian J Dermatol.

[46] Rengasamy M, Chellam J, Ganapati S (2017). Systemic therapy of dermatophytosis: practical and systematic approach. Clin Dermatol Rev.

[47] Jensen JM, Pfeiffer S, Akaki T, Schröder JM, Kleine M, Neumann C, Proksch E, Brasch J (2007). Barrier function, epidermal differentiation, and human beta-defensin 2 expression in tinea corporis. J Invest Dermatol.

[48] Shi TW, Zhang JA, Tang YB, Yu HX, Li ZG, Yu JB (2015). A randomized controlled trial of combination treatment with ketoconazole 2% cream and adapalene 0.1% gel in pityriasisversicolor. J Dermatolog Treat..

[49] Baran R, Hay RJ, Garduno JI (2008). Review of antifungal therapy, part II: treatment rationale, including specific patient populations. J Dermatolog Treat.

[50] Maulingkar SV, Pinto MJ, Rodrigues S (2014). A clinico-mycological study of dermatophytoses in Goa, India. Mycopathologia.

[51] Pilmis B, Jullien V, Sobel J, Lecuit M, Lortholary O, Charlier C (2015). Antifungal drugs during pregnancy: an updated review. J Antimicrob Chemother.

[52] Murase JE, Heller MM, Butler DC (2014). Safety of dermatologic medications in pregnancy and lactation. Part I Pregnancy J Am Acad Dermatol.

[53] Patel VM, Schwartz RA, Lambert WC (2017). Topical antiviral and antifungal medications in pregnancy: a review of safety profiles. J Eur Acad Dermatol Venereol.

[54] Narang T, Mahajan R, Dogra S (2017). Dermatophytosis: fighting the challenge: conference proceedings and learning points. September 2-3, 2017, PGIMER, Chandigarh, India. Indian Dermatol Online J.

[55] Bhatia VK, Sharma PC (2014). Epidemiological studies on Dermatophytosis in human patients in Himachal Pradesh, India. Springer Plus.

[56] Kucheria M, Gupta SK, Chhina DK, Gupta V, Hans D, Singh K (2016). Clinico-mycological profile of Dermatophytic infections at a tertiary care hospital in North India. Int J Com Health and Med Res.

[57] Putta SD, Kulkarni VA, Bhadade AA, Kulkarni VN, Walawalkar AS (2016). Prevalence of dermatophytosis and its spectrum in a tertiary care hospital, Kolhapur. Indian J Basic Appl Med Res.

[58] Ramaraj V, Vijayaraman RS, Rangarajan S, Kindo AJ (2016). Incidence and prevalence of dermatophytosis in and around Chennai, Tamilnadu, India. Int J Res Med Sci.

